# Serum glycopattern and *Maackia amurensis* lectin-II binding glycoproteins in autism spectrum disorder

**DOI:** 10.1038/srep46041

**Published:** 2017-05-09

**Authors:** Yannan Qin, Yanni Chen, Juan Yang, Fei Wu, Lingyu Zhao, Fuquan Yang, Peng Xue, Zhuoyue Shi, Tusheng Song, Chen Huang

**Affiliations:** 1 Department of Cell Biology and Genetics, Environment and Genes Related to Diseases Key Laboratory of Education Ministry, School of Basic Medical Sciences, Xi’an Jiaotong University Health Science Center, Xi’an 710061, P. R. China; 2 Xi’an Child’s Hospital of Medical College of Xi’an Jiaotong University, Xi’an Child’s Hospital, Xi’an 710002, P. R. China; 3 Laboratory of Proteomics, Institute of Biophysics, Chinese Academy of Sciences, Beijing 100101, P. R. China; 4 The Department of Biology, College of Liberal Arts and Science, The University of Iowa, Iowa 430015, USA

## Abstract

The pathophysiology of autistic spectrum disorder (ASD) is not fully understood and there are no diagnostic or predictive biomarkers. Glycosylation modified as many as 70% of all human proteins can sensitively reflect various pathological changes. However, little is known about the alterations of glycosylation and glycoproteins in ASD. In this study, serum glycopattern and the *maackia amurensis* lectin-II binding glycoproteins (MBGs) in 65 children with ASD and 65 age-matched typically developing (TD) children were compared by using lectin microarrays and lectin-magnetic particle conjugate-assisted LC-MS/MS analyses. Expression of Siaα2-3 Gal/GalNAc was significantly increased in pooled (fold change = 3.33, *p* < 0.001) and individual (*p* = 0.009) serum samples from ASD versus TD children. A total of 194 and 217 MGBs were identified from TD and ASD sera respectively, of which 74 proteins were specially identified or up-regulated in ASD. Bioinformatic analysis revealed abnormal complement cascade and aberrant regulation of response-to-stimulus that might be novel makers or markers for ASD. Moreover, increase of APOD α2-3 sialoglycosylation could sensitively and specifically distinguish ASD samples from TD samples (AUC is 0.88). In conclusion, alteration of MBGs expression and their sialoglycosylation may serve as potential biomarkers for diagnosis of ASD, and provide useful information for investigations into the pathogenesis of ASD.

Autism spectrum disorder (ASD) is a highly heritable complex neurodevelopmental disorder characterized by compromised social communication and interaction^1^. Epidemiologic studies indicate that ASD is dramatically increasing each year all over the world with social, behavioural and economical burdens^2^,^3^. Although ASD symptoms begin in infancy, diagnosis is dependent entirely on the recognition of the cardinal behavioral signs that are present by at least 3 years of age^4^, which makes behavioral intervention less effective and can generate false positive identification^5^,^6^. The genetic architecture of ASDs is highly heterogeneous^7^, which refers to chromosomal alterations (e.g., 15q11–q13 duplications)^8^, mutations of single genes (e.g., FMR1 and MECP2)^9^, rare gene mutations (e.g., NLGN3 and SHANK3)^10^, and copy number variation^11^. Because of these, the isolation of specific risk genes for ASD is difficult, and in only a minority of ASD cases can a genetic defect be unequivocally linked to the disorder. Developing serum-based biomarkers, with measurable parameters, is urgently needed to facilitate earlier and more reliable diagnoses.

Proteomic tools allow for an automated, technology-driven large-scale mode of examination provide the chance to determine the whole proteome in a given body fluid without prior assumptions about candidate molecules^12^. Based upon this, a total of five peptide components corresponding to four known proteins [Apolipoprotein (apo) B-100, Complement Factor H Related Protein (FHR1), Complement C1q, and Fibronectin 1 (FN1)] were found greater for autism compared to controls^13^. Three potential biomarker peaks showed m/z ratios of approximately 4.40, 5.15 and 10.38 kDa significantly differentiated the ASD sample from the control group by analyzing whole proteins not peptides after tryptic digestion^14^.

Protein glycosylation, as the most common form of posttranslational modification, with as many as 70% of all human proteins estimated glycosylated^15^, can better and more sensitively reflect the body inflammation^16^, cancer^17^,^18^, diabetes^19^, asthma^20^, and some physiological changes^21^, due to the structural diversity, micro-heterogeneity, and variability^22^. For example, serum alpha-fetoprotein (AFP) has long been used as a diagnostic marker for hepatocellular carcinoma (HCC), however the value of AFP in HCC diagnosis has recently been challenged due to its significant rates of false positive and false negative findings. To improve the efficacy of AFP as HCC diagnostic marker, seven glycoforms from purified serum AFP were identified and it was found that HCC-associated isoforms are all characterized by being mono-sialylated, whereas those associated with benign liver disease are di- sialo species^23^. Recent studies demonstrate that alterations in protein glycosylation tightly correlated with neurological and developmental deficiencies^24^,^25^,^26^. A further study revealed that four copy number variations containing the genes *B3GALT6, GCNT2, LARGE*, and *GALNT9,* and three single genes *B4GALT1, ARSA*, and *GALNTL5,* known to participate in protein glycosylation, are associated with non-complex-autism^27^. However, little is known about the alterations of glycoproteins glycosylation in serum from patients with ASD compared to the healthy volunteers, which might be significant for finding novel biomarkers, pathogenesis, and therapeutic strategies in ASD.

Lectins are carbohydrate-binding proteins that discriminate glycans on the basis of subtle differences in structure. Lectin microarrays enable the simultaneous quantitative analysis of N- and O-linked glycans recognized by various lectins in intact biological samples without the need for glycan release^28^,^29^. Glycoprotein enrichment through lectin affinity coupled with advanced liquid chromatography-tandem mass spectrometry (LC-MS/MS) are useful tools for identification of targeted peptide sequence^30^,^31^. This study mainly compared glycopattern and the *maackia amurensis* lectin-II binding glycoproteins (MBGs) in serum samples from 65 children with ASD and 65 age-matched typically developing (TD) children by using lectin microarrays and lectin-magnetic particle conjugate-assisted LC-MS/MS analyses. The bioinformatic analysis was further utilized to reveal the biological functions of these MBGs in ASD. The lectin/glyco-antibody microarray (LGAM) was designed for validation of α2–3 sialoglycosylation of MBGs in individual serum samples and evaluation of the diagnosibility. The integrated strategy is summarized in Fig. 1.

## Results

### Alteration of Glycopattern in Sera from ASD versus TD

**Figure 2:**
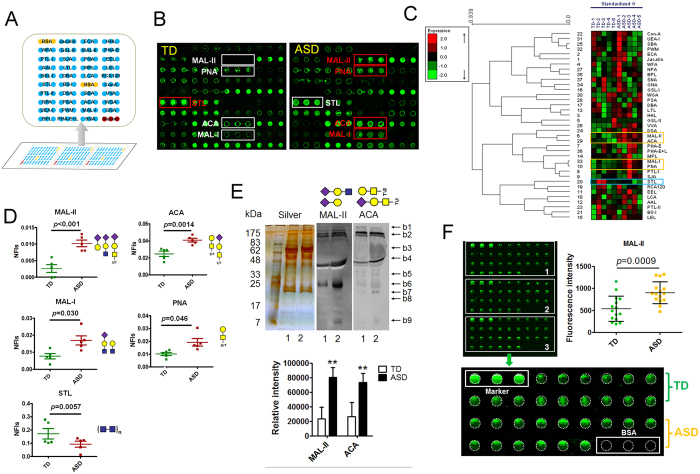
Changes in glycopatterns of sera from autism spectrum disorder (ASD) and age-matched typically developing (TD) children by lectin microarrays. (**A**) Layout of the lectin microarray. (**B**) Images of Cy3-labeled sera proteins from TD and ASD bound to the lectin microarrays. Fluorescent images were scanned with a 70% photomultiplier tube and 100% laser power settings in a Genepix ^40^00B confocal scanner. A portion of the slide with three replicate lectin arrays is shown. The lectins exhibited significant differences, marked with white frames. (**C**) Hierarchical clustering analysis of NFIs for the 37 lectins from TD-1~5 and ASD-1~5. The samples are listed in columns, and lectins are listed in rows. The color and intensity of each square indicates expression levels relative to the other data in the row. Red, high; green, low; black, medium. Yellow and blue frame marked higher and lower binding intensity of lectin in ASD vs. TD sera. (**D**) Differential analysis of NFIs for five lectins from TD-1~5 and ASD-1~5. NFIs of each lectin for the TD and ASD groups were compared according to the following criteria: fold change ≥1.5 or ≤0.67 indicated up-regulation or down-regulation. Differences for each lectin between TD and ASD groups were further tested by Paired student’s t-test using SPSS Statistics 19 (*p* < 0.05). (**E**) Binding patterns of glycoproteins in pooled sera from TD (*n* = 50, lane 1) and ASD (*n* = 50, lane 2) samples for MAL-II (middle) and ACA (right). Gels were stained directly with alkaline silver as control (left). The average gray value for each lane was from Image pro-Plus 6.0 analysis and compared between two groups (*P < 0.05, **P < 0.01, and ***P ≤ 0.001). (**F**) Validation of Siaα2-3 Gal/GalNAc expression in individual serum samples using serum micorarrays. Fluorescent images were scanned with the 50% photomultiplier tube and 100% laser power settings using a LuxScan 10 K Microarray Scanner. Scatter plot analysis of the original data achieved from the serum microarrays. Statistical significance of differences between groups was indicated by the p-value.

The layout of the lectin microarray, and the resulting glycopatterns of serum glycoproteins defined by the microarrays for the ASD and TD groups are shown in Fig. 2A,B. The original data were imported into EXPANDER 6.0 for hierarchical clustering analysis (Fig. 2C). The normalized fluorescent intensities (NFIs) and the sugar-binding specificities for each of the 37 lectins from the two groups are summarized in Table S1. As a result of differential analysis, five lectins showed significant differences between ASD and TD groups. MAL-II (Siaα2-3 Gal/GalNAc) and MAL-I (Siaα2-3Galβ-1,4GlcNAc and Galβ-1,4GlcNAc) showed the most significantly increased NFIs (fold change = 3.33 and 2.20, *p* < 0.001 and *p* = 0.030), and ACA and PNA (Galβ1-3GalNAcα-Ser/Thr (T) and sialyl-T(ST)) showed also significantly increased NFIs (fold change = 1.65 and 1.90, *p* = 0.0014 and 0.046) in the ASD versus the TD group (Fig. 2D). However, STL (trimers and tetramers of the GlcNAc) showed significantly decreased NFIs (fold change = 0.54, *p* = 0.0057) in the ASD versus the TD group (Fig. 2D). To validate the different abundance of certain kinds of glycans between ASD and TD, the lectin blotting was performed with MAL-II and ACA in the pooled TD (n = 50, lane 1) and ASD (n = 50, lane 2) sera. The result of SDS-PAGE showed that sera proteins from ASD and TD were similar in their molecular weight. The lectin blotting analysis showed a total of nine apparent bands (b1–b9) and several minor bands belonging to different molecular weight ranging from 7 to 175 kDa (Fig. 2E). Similarly but differently, MAL-II and ACA showed stronger binding to glycoprotein bands (b2, b4, b6, and b9 for MAL-II, b3, b4, b6, b7, and b9 for ACA) in the ASD than the TD sera. In addition, the results of serum microarray revealed that expression of Siaα2-3 Gal/GalNAc recognized by MAL-II was significantly increased (*p* = 0.0009) in individual serum samples from ASD versus TD, which were coincident with the results of the lectin microarrays (Fig. 2F).

### Identification of MBGs

**Figure 3:**
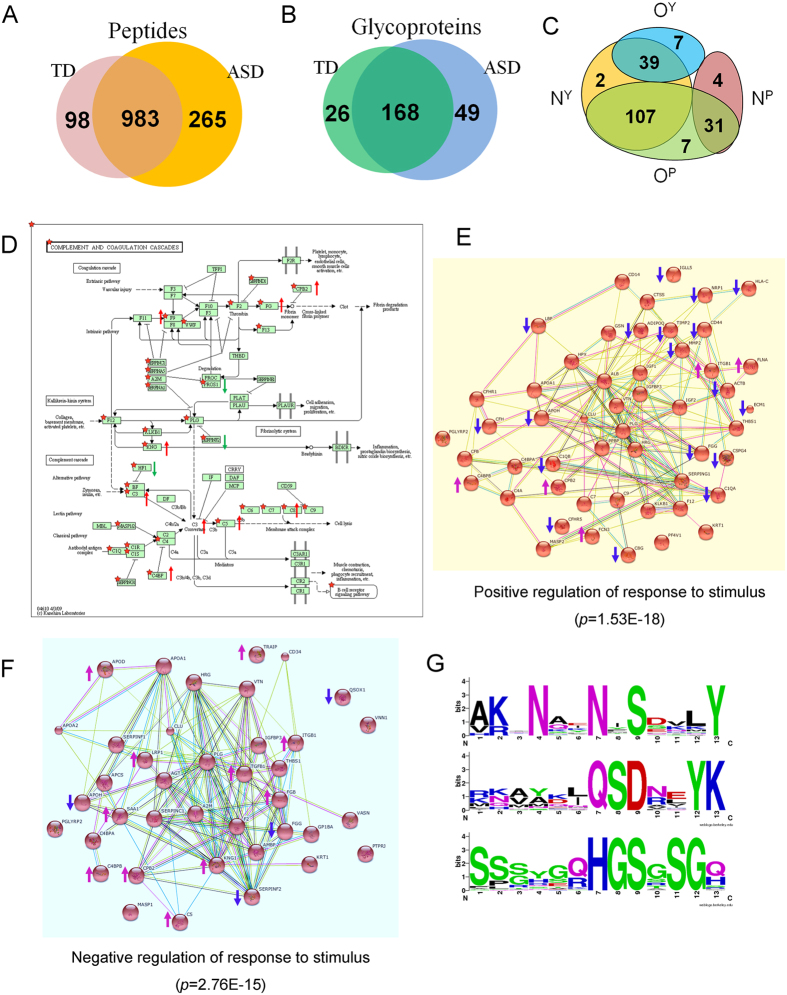
Characterization and bioinformatic analysis of MAL-II binding glycoproteins (MBGs). (**A,B**) Identification of peptides and their corresponding glycoproteins in TD and ASD sera by LC-MS/MS. (**C**) Proportion of known N-glycoproteins (N^Y^) and O-glycoproteins (O^Y^) by UniProtKB/Swiss-Prot database and the predicted glycoproteins with potential N-glycosylation sites (N^P^) and potential O-glycosylation sites (O^P^). (**D**) KEGG pathway analysis of the identified MBGs (marked with a red star) in complement and coagulation cascades^60^. Red arrow, up-regulation of MBGs; green arrow, down-regulation of MBGs in ASD. Protein interaction network analysis of the identified MBGs (red sphere) that were up-regulated (red arrow) or down-regulated (blue arrow) in positive regulation (**E**) and negative regulation (**F**) of response-to-stimulus processes in ASD sera. (**G**) Possible N-glycosylation and O-glycosylation motifs around asparagine and serine residues for the α2-3-linked sialylated glycopeptide domain. WebLogo generated relative frequency plots of the significant sequence motif. The heights of the residues are approximately proportional to their binomial probabilities.

Because of the significant increase of Siaα2-3 Gal/GalNAc expression (α2-3 linked sialoglycosylation) on glycoproteins in sera from ASD versus TD groups, two new questions were then raised: firstly, whether the Siaα2-3 Gal/GalNAc or the α2-3 linked sialoglycosylated proteins were increased, and secondly, what kinds of proteins were α2-3 linked sialoglycosylated and what are their potential biological functions in ASD sera. To solve these problems, MMPCs were applied to isolate MBGs from the pooled sera in two groups. The isolated protein fractions were analyzed by SDS-PAGE (Supplementary Figure S1). The eluted protein factions were slightly darker for ASD (green frame) than TD sera (blue frame). The peptide mixtures were identified in triplicate to reduce variances for individual proteins. A total of 1081 (corresponding 194 glycoproteins) and 1248 (corresponding 217 glycoproteins) unique peptides were identified from TD and ASD sera, respectively (Table S2). Notably, 983 (75.0%) of the peptides, corresponding 168 (69.1%) proteins, were common to both sera, whereas 26 proteins and 49 proteins were specially identified in TD and ASD sera respectively (Fig. 3A,B). By mapping to the UniProtKB/Swiss-Prot database, 213 of the identified proteins were known proteins, of which 146 proteins (68.5%) were known N-glycoproteins (N^Y^) and 45 proteins (21.1%) were O-glycoproteins (O^Y^) (Table S2 and Fig. 3C). Other 90 proteins including 30 unknown proteins (cannot search in UniProt database) and 60 “unproven” proteins (without glycosylation information by UniProt database) were novel identified glycoproteins in the study (Table S2). According to two online glycosylation site prediction servers (NetNGlyc 1.0^32^ and NetOGlyc 4.0^33^), 35 of the 90 proteins were predicted to have potential N-glycosylation sites (N^P^), and 79 were predicted to have potential O-glycosylation sites (O^P^). In addition, 106 known N-glycoproteins were also predicted to have potential O-glycosylation sites (O^P^) (Table S2 and Fig. 3C). There were still seven non-glycoproteins (Table S2). A spectral index (SI) based on the spectra and peptide counts was calculated to compare protein expression level between TD and ASD sera. Totally, 25 proteins (e.g., apolipoprotein D [APOD] and complement component C8 [C8B]) were up-regulated (ratio ≥ 1.5), while 23 proteins (e.g., complement C1q subcomponent subunit A [C1QA] and Neuropilin-1 [NRP1]) were down-regulated (ratio ≤ 0.67) in ASD relative to TD sera (Table S2 and Supplementary Figure S1). Comparing the molecular weights (MW) and isoelectric points (pI) of the MBGs, the majority had MWs lower than 200 kDa and pI values lower than 7 in both TD and ASD sera (Supplementary Figure S1).

### Gene Ontology Analysis of the MBGs

To investigate the major biological functions of the identified MBGs, Blast2GO was applied to analyze them for functional enrichment according to three grouping classifications: cellular components, biological processes, and molecular functions. Of the 243 identified MBGs, 216 gene ontology (GO) annotations were available (Supplementary Figure S1). In the cellular component group, 150 proteins (61.2%) were extracellular region proteins, and 90 proteins (36.7%) were membrane proteins. In biological processes, 152 proteins (62.0%) were involved in single-organism processes and 136 proteins (55.5%) were involved in response-to-stimulus processes. In terms of molecular function, proteins with binding ability formed the largest group (146, 59.6%), and other smaller groups identified included enzyme regulatory activity (38, 15.5%) and catalytic activity (38, 15.5%). To analyze potential differences in the GO annotations between ASD and TD serum samples, GO enrichment analysis was performed. ASD serum samples were enriched versus TD serum samples in annotations including: fibrinogen complex, lyase, transposase, chromosome segregation, and maintenance of location in cell. On the other hand, ASD serum samples were depleted versus TD serum samples in annotations including: cell leading edge, nucleoside-triphosphatase regulator, peptide binding, and developmental growth (Supplementary Figure S1).

### KEGG Pathway and Protein Interaction Network Analysis

In total, 184 of 243 identified MBGs were annotated in DAVID Bioinformatics Resources (version 6.7). These MBGs were mapped to 6 KEGG pathways with thresholds of count ≥5 and a *P*-value < 0.05 versus the background signal of the human genome; the identified KEGG pathways included complement and coagulation cascades, systemic lupus erythematosus, ECM-receptor interaction, and others (Table S3). A total of 38 MBGs were involved in complement and coagulation cascades, of which most MBGs (e.g., carboxypeptidase B2 [CPB2], kininogen-1 [KNG1], and complement C5 [C5]) were up-regulated except that vitamin K-dependent protein S (PROS1), alpha-2-antiplasmin (SERPINF2), and complement factor H (CFH) were down-regulated in ASD sera compared to TD sera (*p* = 3.79E-54) (Fig. 3D). In addition, 217 matched MBGs were queried against the STRING *Homo sapiens* database to determine their functional relevance. Through enrichment analysis of biological processes, 18 versus 5 of the 49 proteins responsible for positive regulation of response to stimulus (*p* = 1.53E-18) exhibited decreased versus increased expression (Fig. 3E), meanwhile 11 versus 4 of the 39 proteins responsible for negative regulation of response to stimulus processes (*p* = 2.76E-15) showed increased versus decreased expression (Fig. 3F) in ASD sera.

### Sequence Motif Preference of MBGs

Typically, N-glycosylation occurs at N-X-S/T motifs (where X cannot be proline) in mammals. Our data set provided a good basis to test the generality of this motif and to identify further consensus sequences. Notably, 12 specific nonredundant consensus sequences with a high motif score and fold increase >30 were identified (Supplementary Figure S1). The position-specific amino acid frequencies of the surrounding asparagine residues (13 amino acids to both termini) were compared, and the motif [AVH][KR]xNxxNxSxxxY (where “x” denotes any residue, [AVH] and [KR] represent several amino acid residues that might appear in the position, and the bullet point denotes a possible glycosite) was identified as a possible N-glycosylation motif around asparagine (Fig. 3G). Interestingly, xxxxxxQSDxxYK and xxxxxxHGSxSGx motifs were significantly overrepresented (fold increase = 81.62 and 67.07) in the MBG data (Fig. 3G), which might represent O-linked glycosylation motifs around serine residues for the α2-3-linked sialylated glycopeptide domain. However, to further confirm the O-glycosites in MBGs, it still needs much more in-depth studies.

### Expression and Sialoglycosylation of MBGs in Individual Serum Samples

**Figure 4:**
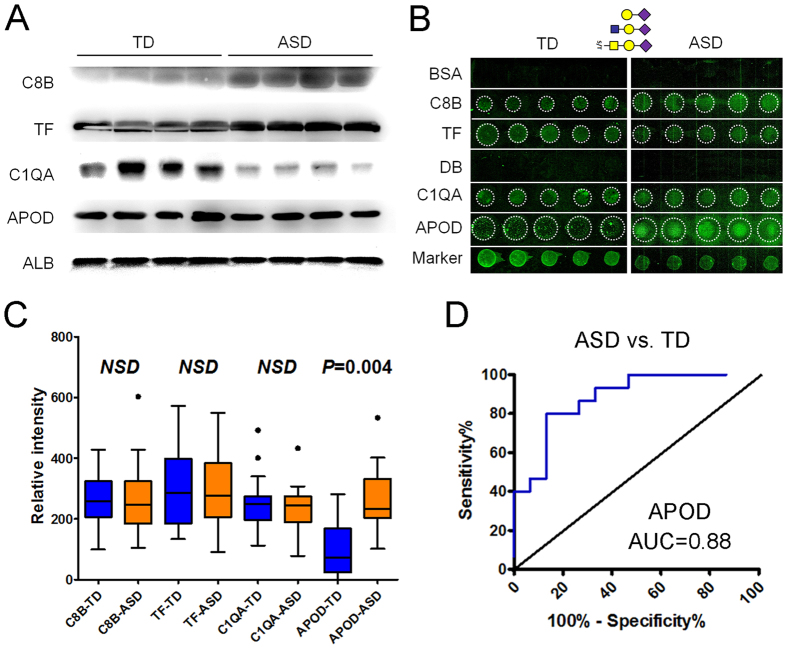
Validation of the expression and sialoglycosylation of the MBGs in individual serum samples. (**A**) Western blot analysis of the expression of C8B, TF, C1QA, and APOD in four TD and four ASD serum samples. (**B**) Scan images derived from the LGAMs for TD and ASD sera. (**C**) Box plot analysis of binding intensities for C8B, TF, C1QA, and APOD from 30 cases of TD and ASD serum by the LGAMs. Error bars represent 95% confidence intervals for means. Statistically significant differences between groups are indicated by the *P*-values. (**D**) ROC curve analysis of the α2-3 sialoglycosylated APOD for differentiating ASD samples from TD samples.

A western blot was performed to verify the expression of C8B, serotransferrin (TF), C1QA, and APOD in individual serum samples. As a result, expression of C8B and TF were increased and expression of C1Q was decreased in four tested ASD samples compared to four TD samples, which were consistent with results of MS (Fig. 4A). However, expression of APOD was not significantly different between TD and ASD samples (Fig. 4A). LGAMs were designed to detect α2-3 linked sialoglycosylation of C8B, TF, C1QA, and APOD in 15 TD and 15 ASD individual serum samples (Fig. 4B). As a result, no significant differences for C8B, TF, and C1QA sialoglycosylation were detected between two groups. However, α2-3 siologlycosylation of APOD was significantly increased in ASD samples relative to TD samples (*p* = 0.004) (Fig. 4C). ROC curve analysis revealed that serum levels of α2-3 sialoglycosylated APOD resulted in an AUC of 0.88, with a specificity of 86.7% and a sensitivity of 80.6% for differentiating ASD from TD) (Fig. 4D).

## Discussion

Lectins are carbohydrate-binding proteins that are neither antibodies nor enzymes, which have a wide range of glycan-binding specificities. These characteristics make them suitable for the characterization of a glycome of cell, tissue, serum, saliva, and so on. In this study, expression of Siaα2-3 Gal/GalNAc (MAL-II) showed the most significant increase (fold change = 3.3, *p* < 0.001) in sera from ASD versus TD (Fig. 2D), and therefore, the intriguing MBGs were further captured by MMPCs from ASD and TD sera and identified by LC-MS/MS. MAL-II is a known glycoprotein, so it was possible that a few glycan-recognition proteins bound to glycans on MAL-II be pulled down together using the MMPCs. This factor, together with nonspecific protein adsorption, resulted in the identification of “non-glycoproteins” by MS (Table S2). To eliminate the impact of these two problems as effectively as possible, an optimum binding buffer conducive to high affinity interactions between the carbohydrate-binding domain of MAL-II and α2-3 linked sialic acids on MBGs was used, together with appropriate denaturing by washing with 0.1% (v/v) Tween 20 several times.

There was a notable phenomenon that the binding patterns of MAL-II and ACA to the glycoproteins in sera of ASD and TD were extremely similar according to lectin blotting (Fig. 2E). Now that MAL-II is proven to bind specifically to α2-3 sialic acid on T antigen^34^, it could be speculated that increased NFIs of MAL-II bound to ASD sera vs. TD sera was actually resulted from higher expression of α2-3 sialosyl-T antigens on MBGs. In the subsequent protein identification and characterization, 46 of 243 MBGs were known O-linked glycoproteins, and 145 MBGs were potential O-glycosylated proteins. Sequence motif preference analysis also indirectly indicated that xxxxxxHGSxSGx and xxxxxxQSDxxYK motifs surrounding serine residues significantly overrepresented in MBGs might be potential O-linked glycosylation motifs (Fig. 3G). A previous study found that no changes in the plasma N-glycome were associated with ASD using hydrophilic interaction high performance liquid chromatography^35^, which was complemented in this study. However, although these data provided clues for predicting glycan structures of MBGs, the fact still needed to be experimentally proved by possibly employing advanced glycomic techniques, such as matrix-assisted laser desorption ionization time-of-flight mass spectrometry^36^.

Recent interest in profiling the glycome stems from the potential of glycans as disease markers^37^,^38^. With glycans as disease markers there are several intrinsic advantages compared to other biomolecules, specifically proteins: (1) glycan biosynthesis is more significantly affected by disease states than protein production. (2) Aberrant glycosylation can potentially affect nearly every glycoprotein produced in the diseased cell. (3) Given the current technology, it is far simpler to quantitate oligosaccharide expression than protein expression^39^. Analysis of glycan on protein involves several levels of complexity, which includes simple compositional profile, glycan structure, protein-specific glycosylation, and the site-specific glycosylation (with increasing complexity)^38^. In this study, the glycopattern was detected firstly by using lectin microarrays, and then the glycan associated proteins were further isolated and identified based upon LC-MS/MS. It is obvious that even the same glycan on various glycoproteins may play different roles that depend on functions of glycoproteins themselves in disease^40^. Therefore, this study revealed not just the altered glycans but also the glycan associated proteins so as to demonstrate the capacity of altered glycosylation of protein as biomarkers for ASD diagnosis and provide further information for investigations into the mechanisms of ASD. Previous studies showed that human serum N-glycan profiles are age and sex dependent^41^. In this study, to minimize the effects of inter- and intra- patient variations, 65 children with ASD and 65 age-matched TD children with similar sex ratio were enrolled. Then, 50 TD and 50 ASD serum samples were pooled respectively for lectin microarray and LC-MS/MS identification, and other 15 TD and 15 ASD samples maintained individually were used for LGAMs detection.

Many researchers have repeatedly described immune dysfunction in ASD, symptoms of which include neuroinflammation, the presence of autoantibodies, increased T cell responses, and enhanced innate NK cell and monocyte immune responses^42^. The complement system is a part of the immune system that helps or complements the ability of antibodies and phagocytic cells to clear pathogens from an organism^43^. In recent years, studies have shown that complement cascade, a major effecter arm of the innate immune system, is almost certainly involved in synaptic remodeling by tagging destined neurons and synapses for destruction^44^. In addition, developing astrocytes release signals that induce the expression of complement components in the central nervous system (CNS). In the mature brain, early synapse loss is a hallmark of several neurodegenerative diseases. Complement proteins are profoundly upregulated in many CNS diseases prior to signs of neuron loss^45^. Therefore, the abnormal complement cascade displayed in this study might be a pivotal manifestation mode of immune dysfunction in ASD (Fig. 3D). Besides, this study found that almost all MBGs (e.g., NRP1 and CFHR5) responsible for positive regulation of response-to-stimulus processes were down-regulated, and most MBGs (e.g., FGB and APOD) responsible for negative regulation of response-to-stimulus processes were up-regulated in the ASD sera (Fig. 3E,F), which might be one important maker or marker for ASD, and provide useful information for further in-depth investigations of the pathogenesis and treatment of ASD.

Typically, sialic acid is found as a component of the oligosaccharide chains of mucins, glycoproteins, and glycolipids occupying terminal, nonreducing positions of N- or O-glycans. Sialic acid levels in serum are associated with liver diseases^46^, rheumatic diseases^47^, and type-2 diabetes^48^. In this study, western blot analysis validated the alteration of C8B, TF, and C1QA expression, but not APOD expression, in individual ASD and TD serum samples (Fig. 4A). LGAMs revealed significantly increased expression of α2-3 sialoglycosylation of APOD in individual ASD serum samples, which greatly explained the no difference in expression of APOD protein between ASD and TD sera, and emphasized that both MBGs and their α2-3 sialoglycosylation were associated to ASD. ROC curve analysis noted that sialoglycosylated APOD could sensitively and specifically distinguish ASD from TD children as candidate biomarkers (AUC = 0.88), and indicated the importance and necessary of studying the alteration of glycoproteins glycosylation in sera for diagnose of ASD.

In conclusion, expression of α2-3 sialosyl-T antigens was significantly increased in sera of ASD versus TD. A total of 194 and 217 MBGs were identified from TD and ASD sera respectively, of which 74 proteins were specially identified or up-regulated in ASD sera. Bioinformatic analysis revealed that abnormal complement cascade and aberrant cellular regulation of response-to-stimulus might be novel makers or markers for ASD, which provide novel information for further in-depth investigations into the pathogenesis of ASD. More importantly, LGAMs revealed significantly increased expression of α2-3 sialoglycosylation of APOD in individual ASD serum samples, which might serve as potential biomarkers for diagnosis of ASD.

## Materials and Methods

### Study Approval

The collection and use of all human pathology specimens for research presented here were approved by the Ethical Committee of Northwest University, Shaanxi Provincial People’s Hospital and Fourth Military Medical University (Xi’an, China). Written informed consent was received from participants for the collection of their whole saliva and serum. This study was conducted in accordance with the ethical guidelines of the Declaration of Helsinki.

### Subjects

Sixty-five children with ASD and 65 age-matched TD children between 2.5 and 6 years of age were enrolled. Children in ASD group were recruited from Xi’an Children’s Hospital, the First Affiliated Hospital of Xi’an Jiaotong University, and the Second Affiliated Hospital of Xi’an Jiaotong, Xi’an, China. All children with ASD were examined by clinical experts on autism. A developmental behavioral pediatrician and a pediatric neurologist or psychiatrist examined all the children. All consultants agreed on the diagnosis of ASD according to DSM-V criteria^49^. Subjects with tuberous sclerosis complex, Rett syndrome, Prader Willi syndrome, Angelman syndrome, or Fragile X syndrome were excluded. All participants were screened via a parental interview for current and past physical illness. The control group consisted of healthy TD children recruited from the same area to minimize the influence of different environments. Children in both the ASD and TD groups who had any type of infection or disease less than 2 weeks before the time of examination were excluded. Intelligence quotient was measured using the Gesell Development Schedule. ASD was evaluated with the autism diagnostic observation schedule (Table 1 and Table S4).

Approval for this research was obtained from the Ethics Committee and the Human Research Review Committee of Xi’an Jiaotong University (Xi’an, China). All parents of the participants enrolled in the study provided written informed consent. All experiments were carried out in accordance with the approved guidelines.

### Sample Collection and Preparation

All blood samples were collected by a pediatric nurse and venous blood was collected. The blood was allowed to clot at room temperature for 25 min. The clot was then removed by centrifuging at 1, 500 g for 10 minutes in a refrigerated centrifuge. The resulting supernatant is immediately transferred to a clean polypropylene tube added with EDTA-free inhibitor cocktail (Halt protease inhibitor; Thermo Scientific Pierce Protein Research Products, Rockford, IL, USA) at a concentration of 10 μL/mL serum. The produced serum was aliquoted into small portions and immediately frozen on dry ice and stored at −80 °C. To normalize the differences between subjects and to tolerate individual variation, 50 μL of 50 serum samples from TD and ASD groups were pooled respectively for lectin microarray and LC-MS/MS detection. The other 15 samples from each group were maintained individually for further validation.

### Lectin Microarray and Data Analysis

The lectin microarray was produced and incubated with Cy3 fluorescent dye (GE Healthcare) labelled serum proteins according to our previous protocol^50^,^51^,^52^,^53^ that are described in the Supplementary Materials *and Methods* in detail. Fifty TD and 50 ASD serum samples were used for lectin microarray detection. Twenty microliter (20 μL) from each sample and 10 samples in a pool were prepared to form TD-1~5 and ASD-1~5 subgroups. The acquired images were analyzed at 532 nm for Cy3 detection using Genepix 3.0 software. The averaged background was subtracted, and values less than the average background ± 2 standard deviations (SD) were removed from each data point. The median of the effective data point for each lectin was globally normalized to the sum of the median of all effective data points for each lectin in a block. Each sample was observed consistently with three repeated slides, and the normalized median of each lectin from 9 repeated blocks was averaged and the SD determined. Normalized data for the TD and ASD groups were compared according to the following criteria: fold change ≥1.5 or ≤0.67 indicated up-regulation or down-regulation. Differences between the two arbitrary data sets were tested by Paired student’s *t*-test using SPSS Statistics 19. The original data were further analyzed with Expander 6.0 (http://acgt.cs.tau.ac.il/expander/) to perform a hierarchical clustering analysis.

### Serum Microarray and Data Analysis

A serum microarray was produced by using 30 individual serum samples from 15 TD and 15 ASD children each. The Cy3-labeled MAL-II was applied to detect the specific sugar structure in the minimal amount of serum samples that immobilized on the slides according to the fabrication protocol of saliva microarray^51^ with some modifications. Detailed information is provided in the Supplementary Materials and Methods.

### Isolation and Digestion of MBGs

MAL-II-magnetic particle conjugates (MMPCs) were prepared as described^54^,^55^. Two milligrams (~30 μL, measured with Bradford reagent) of protein from pooled TD and ASD sera were incubated with the MMPCs^54^,^55^. The obtained glycoproteins (about 150 μg) were digested by trypsin and PNGase F as described previously^54^,^55^,^56^. Detailed information is provided in the Supplementary Materials and Methods.

### LC-MS/MS Analysis

MS analysis was performed using an LTQ Orbitrap XL mass spectrometer (Thermo Scientific). The detailed parameters used in this experiment are provided in the Supplementary Materials and Methods. The raw data was processed using Proteome Discoverer (version 1.4.0.288, Thermo Fischer Scientific). The MS/MS spectra were searched with SEQUEST engine against the UniProt human complete proteome database and contaminant database (Release 2013_06, 88913 Protein sequences). The search was performed with the following parameters: precursor mass tolerance 20 ppm; MS/MS mass tolerance 0.6 Da; two missed cleavage for tryptic peptides; variable modifications oxidation (M), Methylthio (C), Peptide spectral matches (PSM) were validated by a targeted decoy database search (FDR ≤ 0.01).

### Label-Free Relative Quantification by Spectral Index Calculation

After peptide identification, an algorithm similar to the ProteinExtractor in ProteinScape, which uses a given minimal peptide score (minPepScore) and minimal peptide count per protein (minNrPeps), was applied as described^57^. Among the listed proteins, every peptide spectrum match (PSM) was extracted. A spectral index (SI) based on spectral and peptide counts was calculated as described previously^58^. The raw spectral counts for identified proteins were normalized using the following formulas (Formula 1 and Formula 2):


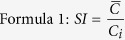



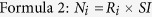


where C_i_ is the total spectral count of run i; and 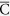 is the averaged total spectral count of all the runs under comparison; N_i_ and R_i_ are the normalized and raw spectral counts of a protein in run i, respectively. The SI, 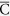/C_i_, was used to normalize the total spectral count of each run to reduce run-to-run variability.

### Data Mining and Bioinformatics

Data analysis and professional softwares used in this study are described in the Supplementary Materials and Methods in detail.

### Lectin/Glyco-Antibody Microarrays and Data Analysis

The lectin/glyco-antibody microarrays (LGAMs) analysis was designed as described^55^,^59^ previously with some modifications. Briefly, rabbit polyclonal antibodies for human C8B, TF, C1QA, and APOD were spotted onto the homemade epoxysilane-coated slides with Stealth microspotting pins (SMP-10B) (TeleChem; Atlanta, GA) using a Capital Smart Arrayer (CapitalBio; Beijing, China). Dilution buffer and BSA were negative controls. Each antibody was printed in quintuplicate per block with triplicate blocks on one slide. Slides were immobilized in a humidity-controlled incubator at 50% humidity overnight. To prevent subsequent interference from glycans on antibodies, the printed slides were oxidized with 200 mM NaIO_4_ solution at room temperature (18–22 °C) for 30 min in the dark to remove all glycans. Slides were then immersed in 1 mM 4-hydroxybenzhydrazide in dimethylformamide at room temperature for 2 h to derivatize the carbonyl groups. After blocking with 1× Carbo-Free^TM^ Blocking Solution (diluted with PBST) for 1 h, 20 μL of serum sample (diluted 1:10 in Carbo-Free^TM^ Blocking Solution with 1% BSA) was applied to the antibody microarrays and rotated in a humidified chamber at 4 °C overnight. The slide was then rinsed with PBST and PBS to remove unbound proteins, and incubated with Cy3 fluorescent dye labeled MAL-II solution and rotated at 37 °C for 2 h. After a final wash, the slides were dried and scanned with a Genepix 4000B microarray scanner (Axon Instruments, CA, USA). Genepix Pro 3.0 was used to extract the spot data. The average background was subtracted, and values less than the average background ±2 SDs were removed from each data point. The median of the effective data points of each antibody for one sample was calculated. Differences between medians of the TD and ASD groups (n = 15) for each antibody were tested by Paired *t*-test using SPSS Statistics 19. Receiver operating characteristic (ROC) curve analysis was performed to evaluate the potential use of sialoglycosylation of MBGs as biomarkers of ASD.

## Additional Information

**How to cite this article:** Qin, Y. *et al*. Serum glycopattern and *Maackia amurensis* lectin-II binding glycoproteins in autism spectrum disorder. *Sci. Rep.* **7**, 46041; doi: 10.1038/srep46041 (2017).

**Publisher's note:** Springer Nature remains neutral with regard to jurisdictional claims in published maps and institutional affiliations.

## Supplementary Material

Supplementary Information

Supplementary Tables

## Figures and Tables

**Table 1:** Basic characteristics of the participants.

	ASD	TD	p-Value
N	65	65	—
Males, (n) %	38 (58.5)	33 (50.8)	—
Age, years^a^	4.0 (2.5–5.5)	4.5 (2.5–6.0)	0.980
Gesell Development Schedule			
Motor area	90.03 ± 0.70	95.8 ± 17.1	0.254
Adaptive area	65.11 ± 15.01	98.10 ± 8.90	<0.001
Language area	53.00 ± 11.00	99.80 ± 10.10	<0.001
Social area	42.00 ± 8.00	97.50 ± 8.80	<0.001
Autism diagnostic observation schedule			
**A:** language and communication	11.12 ± 4.01		
**B:** reciprocal social interaction	24.06 ± 5.87		
**C:** play	5.0 ± 1.37		
**D:** stereotyped behaviors and restricted interests	7.43 ± 1.15		
**E:** other abnormal behaviors	3.43 ± 0.96		

^a^Median (range).

**Figure 1:**
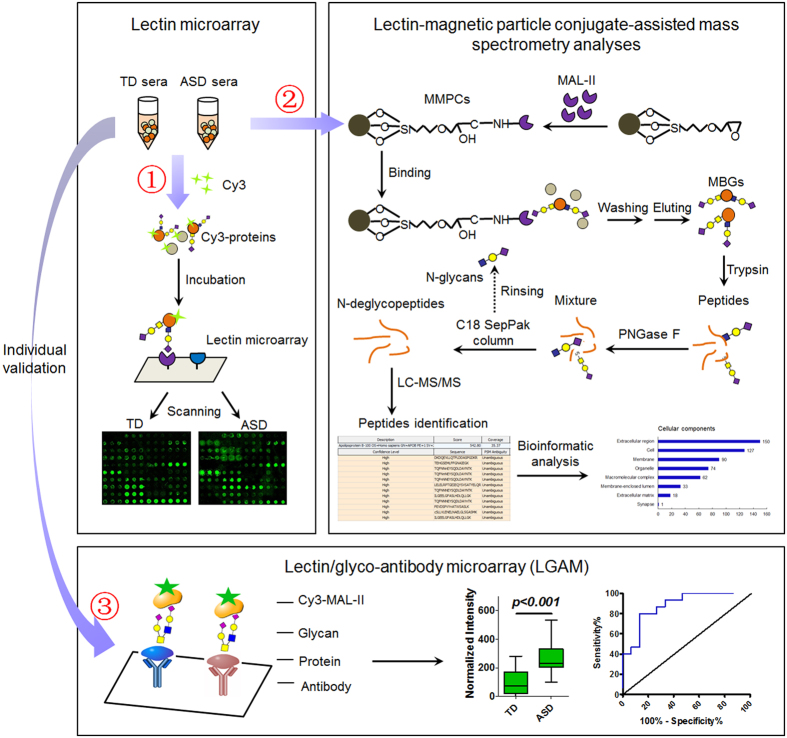
Schematic flow diagram of the integrated strategy used herein.

## References

[b1] Miles J. H. Autism spectrum disorders-a genetics review. Genet. Med. 13, 278–294 (2011). 21358411 10.1097/GIM.0b013e3181ff67ba

[b2] Williams J. G., Higgins J. P. & Brayne C. E. Systematic review of prevalence studies of autism spectrum disorders. Arch Dis. Child 91, 8–15 (2006). 15863467 10.1136/adc.2004.062083 PMC2083083

[b3] Wan Y. . Prevalence of autism spectrum disorders among children in China: a systematic review. *Shanghai Arch*. Psychiatry 25, 70–80 (2013). 10.3969/j.issn.1002-0829.2013.02.003 PMC4054540 24991138

[b4] Hyman, S. *The American Academy of Pediatrics*. http://aapnews.aappublications.org/content/early/2013/06/04/aa pnews.2013604-1 (2013).

[b5] Dawson G. Recent advances in research on early detection, causes, biology, and treatment of autism spectrum disorders. Curr. Opin. Neurol. 23, 95–96 (2010). 20216345 10.1097/WCO.0b013e3283377644

[b6] Zwaigenbaum L. Advances in the early detection of autism. Curr. Opin. Neurol. 23, 97–102 (2010). 20154615 10.1097/WCO.0b013e3283372430

[b7] Devlin B. & Scherer S. W. Genetic architecture in autism spectrum disorder. Curr. Opin. Genet. Dev. 22, 229–237 (2012). 22463983 10.1016/j.gde.2012.03.002

[b8] Betancur C. Etiological heterogeneity in autism spectrum disorders: more than 100 genetic and genomic disorders and still counting. Brain Res. 1380, 42–77 (2011). 21129364 10.1016/j.brainres.2010.11.078

[b9] Yu T. W. & Berry-Kravis E. Autism and fragile X syndrome. Semin. Neurol. 34, 258–265 (2014). 25192504 10.1055/s-0034-1386764

[b10] De Rubeis S. . Synaptic, transcriptional and chromatin genes disrupted in autism. Nature 515, 209–215 (2014). 25363760 10.1038/nature13772 PMC4402723

[b11] Levy D. . Rare de novo and transmitted copy-number variation in autistic spectrum disorders. Neuron. 70, 886–897 (2011). 21658582 10.1016/j.neuron.2011.05.015

[b12] Taurines R. . Proteomic research in psychiatry. J. Psychopharmacol 25, 151–196 (2011). 20142298 10.1177/0269881109106931

[b13] Corbett B. A. . A proteomic study of serum from children with autism showing differential expression of apolipoproteins and complement proteins. Mol. Psychiatry 12, 292–306 (2007). 17189958 10.1038/sj.mp.4001943

[b14] Taurines R. . Serum protein profiling and proteomics in autistic spectrum disorder using magnetic bead-assisted mass spectrometry. Eur. Arch Psychiatry Clin. Neurosci. 260, 249–255 (2010). 19784855 10.1007/s00406-009-0066-5

[b15] Pweiler R., Hermjakob H. & Sharon N. On the frequency of protein glycosylation, as deduced from analysis of the SWISS-PROT database. Biochim. Biophys. Acta 1473, 4–8 (1999). 10580125 10.1016/s0304-4165(99)00165-8

[b16] Lehoux S. . Identification of distinct glycoforms of IgA1 in plasma from patients with immunoglobulin a (IgA) nephropathy and healthy individuals. Mol. Cell Proteomics 13, 3097–3113 (2014). 25071157 10.1074/mcp.M114.039693 PMC4223494

[b17] Liang Y. . Differentially expressed glycosylated patterns of α-1-antitrypsin as serum biomarkers for the diagnosis of lung cancer. Glycobiology 25, 331–340 (2014). 25347993 10.1093/glycob/cwu115

[b18] Croci D. O. . Glycosylation-dependent lectin-receptor interactions preserve angiogenesis in anti-VEGF refractory tumors. Cell 156, 744–758 (2014). 24529377 10.1016/j.cell.2014.01.043

[b19] Lapolla A., Poli T., Valerio A. & Fedele D. Glycosylated serum proteins in diabetic patients and their relation to metabolic parameters. Diabete Metab. 11, 238–242 (1985). 4043491

[b20] El-Seify M. Y., Fouda E. M. & Nabih E. S. Serum level of soluble receptor for advanced glycation end products in asthmatic children and its correlation to severity and pulmonary functions. Clin. Lab. 60, 957–962 (2014). 25016700 10.7754/clin.lab.2013.130418

[b21] Sumer-Bayraktar Z. . Micro- and macroheterogeneity of N-glycosylation yields size and charge isoforms of human sex hormone binding globulin circulating in serum. Proteomics 12, 3315–3327 (2012). 23001782 10.1002/pmic.201200354

[b22] Hart G. W. & Copeland R. J. Glycomics hits the big time. Cell 143, 672–676 (2010). 21111227 10.1016/j.cell.2010.11.008 PMC3008369

[b23] Johnson P. J. . S. K. Structures of disease-specific serum alpha-fetoprotein isoforms. Br. J. Cancer 83, 1330–1337 (2000). 11044358 10.1054/bjoc.2000.1441 PMC2408795

[b24] Freeze H. H., Eklund A. A., Ng B. G. & Patterson M. C. Neurology of inherited glycosylation disorders. Lancet Neurol. 11, 453–466 (2012). 22516080 10.1016/S1474-4422(12)70040-6 PMC3625645

[b25] Drouin-Garraud V. . Neurological presentation of a congenital disorder of glycosylation CDG-Ia: Implications for diagnosis and genetic counseling. Am. J. Med. Genet. 101, 46–49 (2001). 11343337 10.1002/ajmg.1298

[b26] Coman D. . Congenital disorder of glycosylation type 1a: three siblings with a mild neurological phenotype. Clin. Neurosci. 14, 668–672 (2007). 10.1016/j.jocn.2006.04.008 17451957

[b27] Van der Zwaag B. . Gene-network analysis identifies susceptibility genes related to glycobiology in autism. PloS One 4, e5324 (2009). 19492091 10.1371/journal.pone.0005324 PMC2683930

[b28] Yue T. . The prevalence and nature of glycan alterations on specific proteins in pancreatic cancer patients revealed using antibody-lectin sandwich arrays. Mol. Cell Proteomics 8, 1697–1707 (2009). 19377061 10.1074/mcp.M900135-MCP200 PMC2709194

[b29] Fry S. A. . Lectin microarray profiling of metastatic breast cancers. Glycobiology 21, 1060–1070 (2011). 21507904 10.1093/glycob/cwr045

[b30] Kaji H., Yamauchi Y., Takahashi N. & Isobe T. Mass spectrometric identification of *N*-linked glycopeptides using lectin-mediated affinity capture and glycosylation site-specific stable isotope tagging. Nat. Protoc. 1, 3019–3027 (2007). 10.1038/nprot.2006.444 17406563

[b31] Zhang H., Li X. J., Martin D. B. & Aebersold R. Identification and quantification of *N*-linked glycoproteins using hydrazide chemistry, stable isotope labeling and mass spectrometry. Nat. Biotechnol. 21, 660–666 (2003). 12754519 10.1038/nbt827

[b32] Gupta R., Jung E. & Brunak S. Prediction of N-glycosylation sites in human proteins. http://www.cbs.dtu.dk/services/NetNGlyc (2004).

[b33] Steentoft C. . Precision mapping of the human *O*-GalNAc glycoproteome through simple cell technology. EMBO. J. 32, 1478–1488 (2013). 23584533 10.1038/emboj.2013.79 PMC3655468

[b34] Geisler C. & Jarvis D. L. Effective glycoanalysis with *Maackia amurensis* lectins requires a clear understanding of their binding specificities. Glycobiology 21, 988–993 (2011). 21863598 10.1093/glycob/cwr080 PMC3130539

[b35] Pivac N. . Human plasma glycome in attention-deficit hyperactivity disorder and autism spectrum disorders. Mol. Cell Proteomics 10, M110. 004200 (2011). 20974899 10.1074/mcp.M110.004200 PMC3013461

[b36] Willy M. & Jean-Claude M. Analysis of protein glycosylation by mass spectrometry. Nat. Protoc. 2, 1585–1602 (2007). 17585300 10.1038/nprot.2007.227

[b37] Ruhaak L. R., Miyamoto S. & Lebrilla C. B. Developments in the identification of glycan biomarkers for the detection of cancer. Mol. Cell Proteomics 12, 846–855 (2013). 23365456 10.1074/mcp.R112.026799 PMC3617331

[b38] Lebrilla C. B. & An H. J. The prospects of glycan biomarkers for the diagnosis of diseases. Mol. Biosyst. 5, 17–20 (2009). 19081926 10.1039/b811781k

[b39] An H. J., Peavy T. R., Hedrick J. L. & Lebrilla C. B. Determination of *N*-glycosylation sites and site heterogeneity in glycoproteins. Anal. Chem. 75, 5628–5637 (2003). 14710847 10.1021/ac034414x

[b40] Itakura Y. . *N*- and *O*-glycan cell surface protein modifications associated with cellular senescence and human aging. Cell Biosci. 6, 14 (2016). 26893823 10.1186/s13578-016-0079-5 PMC4757982

[b41] Ding N. . Human serum *N*-glycan profiles are age and sex dependent. Age Ageing 40, 568–575 (2011). 21807702 10.1093/ageing/afr084

[b42] Mead J. & Ashwood P. Evidence supporting an altered immune response in ASD. Immunol. Lett. 163, 49–55 (2015). 25448709 10.1016/j.imlet.2014.11.006

[b43] Rus H., Cudrici C. & Niculescu F. The role of the complement system in innate immunity. Immunol. Res. 33, 103–112 (2005). 16234578 10.1385/IR:33:2:103

[b44] Zabel M. K. & Kirsch W. M. From development to dysfunction: Microglia and the complement cascade in CNS homeostasis. Ageing Res. Rev. 12, 749–756 (2013). 23419464 10.1016/j.arr.2013.02.001 PMC3700678

[b45] Stephan A. H., Barres B. A. & Stevens B. The complement system: an unexpected role in synaptic pruning during development and disease. Annu. Rev. Neurosci. 35, 369–389 (2012). 22715882 10.1146/annurev-neuro-061010-113810

[b46] Gruszewska E. . Total and free serum sialic acid concentration in liver diseases. Biomed Res. Int. 2014, 876096 (2014). 24959592 10.1155/2014/876096 PMC4052165

[b47] Chrostek L. . Sialic acid level reflects the disturbances of glycosylation and acute-phase reaction in rheumatic diseases. Rheumatol. Int. 34, 393–399 (2014). 24346772 10.1007/s00296-013-2921-y PMC3925499

[b48] Prajna K. . Predictive value of serum sialic acid in type-2 diabetes mellitus and its complication (nephropathy). J. Clin. Diagn. Res. 7, 2435–2437 (2013). 24392365 10.7860/JCDR/2013/6210.3567 PMC3879886

[b49] American Psychiatric Association. Diagnostic and Statistical Manual of Mental Disorders. 5th Edition. Arlington, VA, 2013.

[b50] Qin Y. . Alteration of protein glycosylation in human hepatic stellate cells activated with transforming growth factor-β1. J. Proteomics 75, 4114–4123 (2012). 22659384 10.1016/j.jprot.2012.05.040

[b51] Qin Y. . Age- and sex-Associated differences in the glycopatterns of human salivary glycoproteins and their roles against influenza A virus. J. Proteome Res. 12, 2742–2754 (2013). 23590532 10.1021/pr400096w

[b52] Zhong Y., Qin Y., Yu H. & Li Z. Avian influenza virus infection risk in humans with chronic diseases. Scientific reports 5, 8971 (2015). 25754427 10.1038/srep08971 PMC4354171

[b53] Zhong Y. . Alteration and localization of glycan-binding proteins in human hepatic stellate cells during liver fibrosis. Proteomics 15, 3283–3295 (2015). 26058380 10.1002/pmic.201500030

[b54] Yang G. . Isolation and identification of native membrane glycoproteins from living cell by concanavalin A-magnetic particle conjugates. Anal. Biochem. 421, 339–341 (2012). 22079135 10.1016/j.ab.2011.10.033

[b55] Yang G. . Selective isolation and analysis of glycoprotein fractions and their glycomes from hepatocellular carcinoma sera. Proteomics 13, 1481–1498 (2013). 23436760 10.1002/pmic.201200259

[b56] Qin Y. . Profiling of concanavalin A-binding glycoproteins in human hepatic stellate cells activated with transforming growth factor-β1. Molecules 19, 19845–19867 (2014). 25460309 10.3390/molecules191219845 PMC6270946

[b57] Kley R. A. . A combined laser microdissection and mass spectrometry approach reveals new disease relevant proteins accumulating in aggregates of filaminopathy patients. Mol. Cell Proteomics 12, 215–227 (2012). 23115302 10.1074/mcp.M112.023176 PMC3536902

[b58] Li Z. . Systematic Comparison of label-free, metabolic labeling, and isobaric chemical labeling for quantitative proteomics on LTQ orbitrap velos. J. Proteome Res. 11, 1582–1590 (2012). 22188275 10.1021/pr200748h

[b59] Li C. . Pancreatic cancer serum detection using a lectin/glyco-antibody array method. J. Proteome Res. 8, 483–492 (2009). 19072160 10.1021/pr8007013 PMC2637303

[b60] Kanehisa M. . KEGG: new perspectives on genomes, pathways, diseases and drugs. Nucleic Acids Res. 45, D353–D361 (2017). 27899662 10.1093/nar/gkw1092 PMC5210567

