# Supplementary material for: Serum glycopattern and Maackia amurensis lectin-II binding glycoproteins in autism spectrum disorder
Source: Sci Rep. 2017 May 9;7:46041. doi: 10.1038/srep46041 (PMC5423032; doi:10.1038/srep46041)
Supplement: Supplementary Information [file srep46041-s1.pdf]

Serum glycopattern and *Maackia amurensis* lectin-II binding glycoproteins in autism spectrum disorder

Yannan Qin[1]†, Yanni Chen[2]†, Juan Yang[1], Fei Wu[1], Lingyu Zhao[1], Fuquan Yang[3], Peng Xue[3], Zhuoyue Shi[4], Tusheng Song[1], Chen Huang[1]*

[1]Department of Cell Biology and Genetics, Environment and Genes Related to Diseases Key Laboratory of Education Ministry, School of Basic Medical Sciences, Xi'an Jiaotong University Health Science Center, Xi'an 710061, P. R. China.

[2]Xi'an Child's Hospital of Medical College of Xi'an Jiaotong University, Xi'an Child's Hospital, Xi'an 710002, P. R. China.

[3]Laboratory of Proteomics, Institute of Biophysics, Chinese Academy of Sciences, Beijing 100101, P. R. China.

[4]The Department of Biology, College of Liberal Arts and Science, The University of Iowa, Iowa 430015, USA.

†These authors contributed equally to this work.

Supplementary Materials and Methods

Lectin microarray and data analysis

Thirty-seven lectins with different binding preferences covering N- and O-linked glycans were spotted onto homemade epoxysilane-coated slides. Each lectin was spotted in triplicate per block with triplicate blocks on one slide. After immobilization, the slides were blocked with blocking buffer containing 2% bovine serum albumin (BSA) in PBS (0.01 mol/L phosphate buffer containing 0.15 mol/L NaCl, pH 7.4) for 1 h, and then washed with PBST (0.2% Tween 20 in PBS) and PBS twice each for 5 min before drying. Eight microgram of sera proteins, labeled with Cy3 fluorescent dye (GE Healthcare) according to a previous protocol[37,38] was diluted in 0.5 mL of incubation buffer containing 2% (w/v) BSA, 500 mmol/L glycine, and 0.1% Tween-20 in PBS. Then the sample was incubated with the blocked lectin microarray at 25 ℃ for 3 h in a rotisserie oven set at 4 rpm. Slides were washed with PBST and PBS twice each for 5 min and dried by centrifugation at 600 rpm for 5 min. The microarrays were scanned with a 70% photomultiplier tube and at a 100% laser power setting with a Genepix 4000B confocal scanner (Axon Instruments, Foster City, CA, USA). The acquired images were analyzed at 532 nm for Cy3 detection using Genepix 3.0 software. The averaged background was subtracted, and values less than the average background ±2 standard deviations (SD) were removed from each data point. The median of the effective data point for each lectin was globally normalized to the sum of the median of all effective data points for each lectin in a block. Each sample was observed consistently with three repeated slides, and the normalized median of each lectin from 9 repeated blocks was averaged and the SD determined. Normalized data for the TD and ASD groups were compared according to the following criteria: fold change ≥1.5 or ≤0.67 indicated up-regulation or down-regulation. Differences between the two arbitrary data sets were tested by Paired student's *t*-test using SPSS Statistics 19. The original data were further analyzed with Expander 6.0 (http://acgt.cs.tau.ac.il/expander/) to perform a hierarchical clustering

analysis.

Serum microarray and data analysis

A serum microarray was produced by using 30 individual serum samples from 15 TD and 15ASD children each. The Cy3-labeled MAL-II was applied to detect the specific sugar structure in the minimal amount of serum samples that immobilized on the slides according to the fabrication protocol of saliva microarray with some modifications. The serum were dissolved in spotting buffer containing 0.5 mg/mL BSA in 1× PBS, pH 7.4, to a concentration of 1 mg/mL and spotted on the epoxysilane-coated slides (CapitalBio, Beijing, China) with Stealth micro spotting pins (SMP-10B) by a PersonalArrayerTM 16 microarrayer (CapitalBio, Beijing, China). Each serum sample was spotted in triplicate. The slides were immobilized in a humidity-controlled incubator at 50% humidity overnight and then dried in vacuum for 3 h at 37 ℃. After immobilization, the slides were blocked with the blocking buffer (3% BSA in 0.01 mol/L PBS, pH 7.4, 0.15 M NaCl, 0.1mM Ca^{++}) for 1 h and rinsed twice with 1× PBS. Then the blocked slide was incubated with Cy3-labeled MAL-II diluted in 0.5 mL of incubation buffer (as the blocking buffer) for 3 h at room temperature in the dark. The slide was washed with 1× PBST twice for 5 min each and washed once with 1× PBS for 5 min, and then dried by centrifugation at 600 rpm for 5 min. The slides were scanned with the 50% photomultiplier tube and 100% laser power settings using a LuxScan 10K Microarray Scanner (CapitalBio, Beijing, China) and the acquired images were analyzed at 532 nm for Cy3 detection by LuxScan10K software. The values less than average background ±2 SD were removed from each data point and the median of the effective data points of each sample was counted. Differences between the arbitrary two groups of medians were tested by Student's t test to each serum samples using SPSS statistics 19.

Selective isolation of MBGs

Two milligrams (~30 μL, measured with Bradford reagent) of protein from pooled TD and ASD sera were

diluted in 600 μL binding buffer (0.1 M Tris-HCl, 150 mM NaCl, 1 mM $CaCl_2$, 1 mM $MgCl_2$, and 1 mM $MnCl_2$, pH 7.4) supplemented with 6 μL proteinase inhibitor cocktail respectively. The MMPCs were rinsed three times with binding buffer, followed by incubation with diluted sera at room temperature for 1 h under gentle shaking. After incubation, the unbound proteins were removed by thoroughly washing three times with a washing buffer (binding buffer supplemented with 0.1% Tween 20). MMPC-bound glycoproteins were eluted with 300 μL elution buffer (0.1% SDS) at room temperature for 30 min under gentle shaking.

Trypsin and PNGase F digestion, peptides collection

The obtained glycoproteins (about 150 μg) were denatured in 8 M urea for 30 min at room temperature. After reduction with 10 mM dithiothreitol and carboxyamidomethylation with 20 mM iodoacetamide, 150 μL proteomics grade trypsin (1:100 w/w of enzyme to protein) in 25 mM NH_4HCO_3 was added and incubated overnight at 37 ℃. The reaction was stopped with 5% glacial acetic acid (5 μL, pH < 2.0). Finally, acid-treated samples were centrifuged at 13 000 × *g* for 10 min and the supernatants were collected and lyophilized. The tryptic peptides were suspended in 100 μL NH_4HCO_3 solution (50 mM, pH 8.0) and incubated with PNGase F overnight at 37 ℃. The reaction was stopped by incubating the solution at 80 ℃ for 5 min. Solutions containing peptides and glycans were lyophilized and resuspended in 100 μL of ultra-pure water. C18 SepPak columns were conditioned by twice washing with acetonitrile (ACN), 0.1% trifluoroacetic acid (TFA) in 50% ACN, and 0.1% TFA, sequentially. The solutions were loaded and pipetted into the column bed. The columns were rinsed three times with 0.5 mL of 0.1% TFA to discard the N-glycans. The peptides were eluted and lyophilized.

LC-MS/MS Analysis

MS analysis was performed using an LTQ Orbitrap XL mass spectrometer (Thermo Scientific). The spray voltage was set at 2.2 kV. Orbitrap spectra (automatic gain control, 1 $\times 10^6$) were collected from 400 to 2000

m/z at a resolution of 60K followed by data-dependent higher-energy collisional dissociation MS/MS (resolution, 7500; collision energy, 45%; activation time, 0.1 ms) of the 10 most abundant ions using an isolation width of 2.0 Da. Charge state screening was enabled to reject unassigned and singly charged ions. A dynamic exclusion time of 35 s was used to discriminate against previously selected ions. The raw data was processed using Proteome Discoverer (version 1.4.0.288, Thermo Fischer Scientific). The MS/MS spectra were searched with SEQUEST engine against the UniProt human complete proteome database and contaminant database (Release 2013_06, 88913 Protein sequences). The search was performed with the following parameters: precursor mass tolerance 20 ppm; MS/MS mass tolerance 0.6 Da; two missed cleavage for tryptic peptides; variable modifications oxidation (M), Methylthio (C), Peptide spectral matches (PSM) were validated by a targeted decoy database search (FDR $\leq$ 0.01).

Data Mining and Bioinformatics

Ontology analysis was performed according to the standard procedure of Blast2GO[1] to gain insights into functional groupings within the set of identified glycoproteins in three aspects of gene ontology (GO). Differences in GO in terms of sera glycoproteins between ASD and TD children were also identified by analysis using WEGO.[2] Biological pathways that were enriched in the identified glycoproteins originated from the KEGG human pathway database.[3] To validate the results of Blast2GO and KEGG, an independent functional enrichment analysis was performed using DAVID (Database for Annotation, Visualization and Integrated Discovery).[4] To determine the functional relevance of the identified glycoproteins, we performed an analysis of protein association networks using STRING 9.05.[5] Finally, the consensus sequence was used to find the characterization of identified proteins using Motif-X (v1.2 10.05.06).[6]

SDS-PAGE, lectin blotting and western blotting

For SDS-PAGE, same amount of protein samples were mixed with 5 × loading buffer and boiled for 4 min at

100 ℃, and then separated on a 10% polyacrylamide resolving gel and a 3% stacking gel. Molecular mass standards (Thermo Scientific) were run for each gel. Gels were then stained directly with alkaline silver. For lectin blotting, the proteins in gels were then transferred to a PVDF membrane (Immobilon-P; Millipore Corp., Bedford, MA) with a wet transfer unit (Hoefer Scientific) for 1.5 h at 32 mA. After transfer, the membranes were washed twice with TTBS (150 mM NaCl, 10 mM Tris-HCl, 0.05% v/v Tween 20, pH 7.5) and then blocked for 1 h with Carbo-Free Blocking Solution (Vector, Burlingame, CA) at room temperature. The membranes were then washed again and incubated with Cy5 (GEHealthcare, Buckinghamshire, U.K.) labeled lectins (2μg/mL in Carbo-Free Blocking Solution) with gentle shaking overnight at 4 ℃ in the dark. The membranes were then washed twice each for 10 min with TTBS and scanned by red fluorescence channel (635 nm excitation/650LP emission) with the voltage of 800 PMT using a phosphorimager (Storm 840, Molecular Dynamics). The gray value was derived from Image pro-Plus 6.0. For western blotting, 30 μg of proteins were separated by 10% SDS-PAGE and then transferred to PVDF membrane. After incubating overnight at 4 ℃ with mouse monoclonal antibodies (GeneTex, diluted 1:1000), membranes were washed and incubated with alkaline phosphatase-conjugated secondary antibodies (GeneTex, diluted 1:5000) for 1 h at room temperature. Relative protein expression was then normalized to albumin levels in each serum sample.

References

1. Conesa, A.; Götz, S.; Garcia-Gomez, J. M.; Terol, J.; Talon, M.; Robles, M. Blast2GO: A universal tool for annotation, visualization and analysis in functional genomics research. *J. Bioinformatics* **2005**, *21*, 3674-3676.

2. Ye, J.; Fang, L.; Zheng, H.; Zhang, Y.; Chen, J.; Zhang, Z.; et al. WEGO: A web tool for plotting GO annotations. *J. Nucl. Acids. Res.* **2006**, *34*, W293-W297.

3. Kanehisa, M.; Goto, S.; Sato, Y.; Furumichi, M.; Tanabe, M. KEGG for integration and interpretation of large-scale molecular datasets. *J. Nucl. Acids. Res.* **2012**, *40*, D109-D114.

4. Huang, D. W.; Sherman, B. T.; Lempicki, R. A. Systematic and integrative analysis of large gene lists using DAVID Bioinformatics Resources. *J. Nat. Protoc.* **2009**, *4*, 44-57.

5. Szklarczyk, D.; Franceschini, A.; Kuhn, M.; Simonovic, M.; Roth, A.; Minguez, P. The STRING database in 2011: Functional interaction networks of proteins, globally integrated and scored. *J. Nucl. Acids. Res.* **2011**, *39*, D561-D568.

6. Chou, M. F.; Schwartz, D. Biological sequence motif discovery using motif-x. *J. Curr. Protoc. Bioinformatics* **2011**, *13*, 15-24.

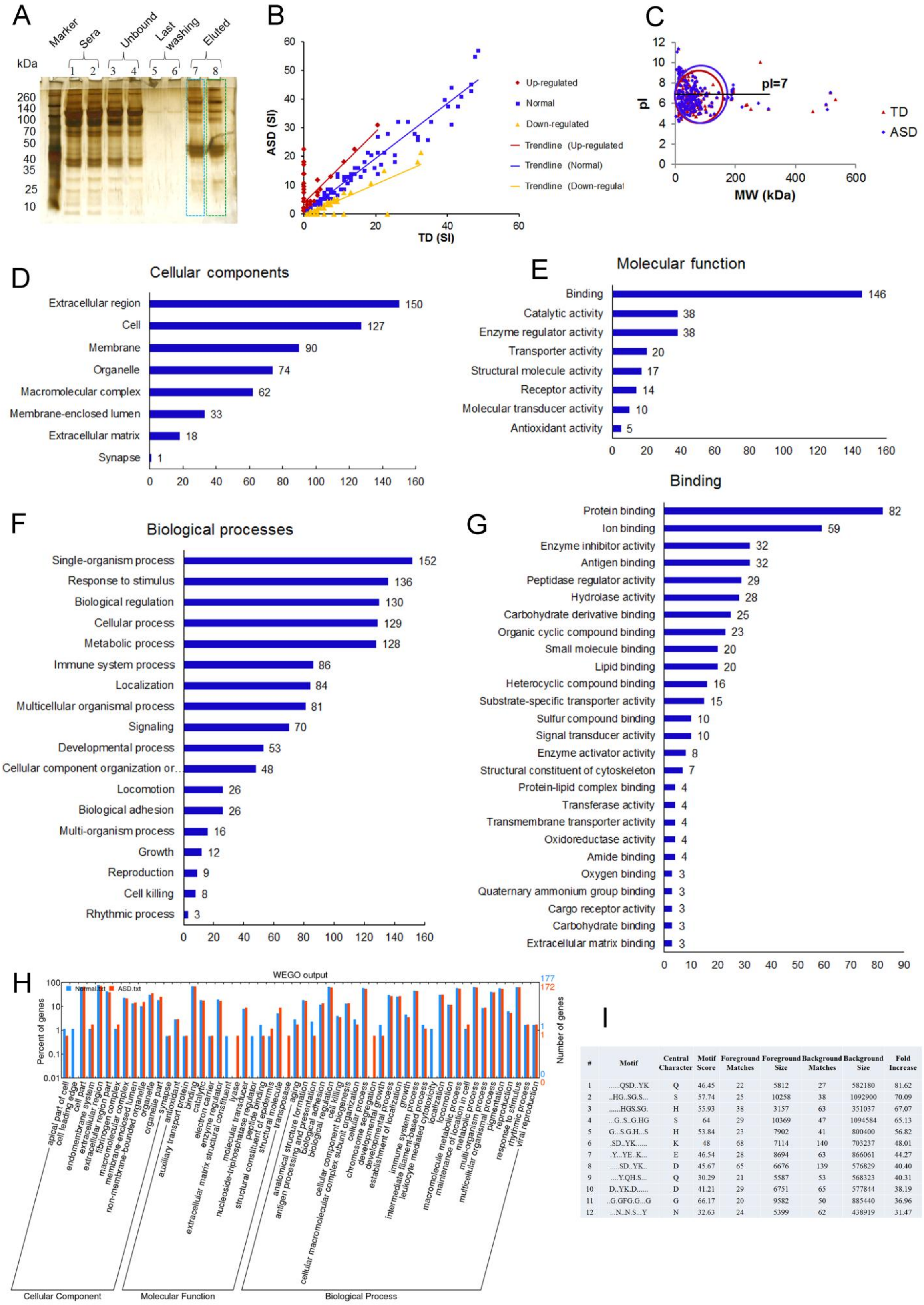

#	Motif	Central Character	Motif Score	Foreground Matches	Foreground Size	Background Matches	Background Size	Fold Increase
1	......QSD..YK	Q	46.45	22	5812	27	582180	81.62
2	..HG..SG.S...	S	57.74	25	10258	38	1092900	70.09
3	......HGS.SG.	H	55.93	38	3157	63	351037	67.07
4	...G..S..G.HG	S	64	29	10369	47	1094584	65.13
5	G...S.G.H...S	H	53.84	23	7902	41	800400	56.82
6	.SD..YK......	K	48	68	7114	140	703237	48.01
7	.Y...YE..K...	E	46.54	28	8694	63	866061	44.27
8	.....SD..YK..	D	45.67	65	6676	139	576829	40.40
9	.....Y.QH.S...	Q	30.29	21	5587	53	568323	40.31
10	D..YK.D......	D	41.21	29	6751	65	577844	38.19
11	..G.GFG.G...G	G	66.17	20	9582	50	885440	36.96
12	...N..N.S...Y	N	32.63	24	5399	62	438919	31.47

Figure S1 – Bioinformatic analysis of MAL-II binding glycoproteins (MBGs). (A) SDS-PAGE analysis of

glycoprotein fractions. Lane 1, TD donor sera; lane 2, ASD sera; lane 3, the unbound fraction of TD sera; lane 4, the unbound fraction of ASD sera; lane 5, the final wash fraction of TD sera; lane 6, the final wash fraction of ASD sera; lane 7, the eluted fraction of TD sera; and lane 8, the eluted fraction of ASD sera. (B) Up-regulation and down-regulation of MBGs expression in ASD sera compared to TD sera. (C) The pI-Mr diagram of the identified MBGs for the two sera pools. (D)-(G) Gene ontology annotations of MBGs according to classifications of cellular component, biological process, molecular function, and binding using Blast2GO software. (H) GO classification and comparison of enrichment of functional groups between ASD and TD was performed using WEGO software. (I) List of specific nonredundant consensus sequences with a high motif score and fold increase >30.

Lectins

Artocapus integrifolia (Jacalin), Erythrina cristagalli (ECA), Hippeastrum Hybrid Lectin (HHL), Wisteria Floribunda Lectin (WFA), Griffonia (Bandeiraea) Simplicifolia Lectin II (GSL-II), Maackia Amurensis Lectin II (MAL-II), Phaseolus vulgaris Agglutinin(E) (PHA-E), Psophocarpus Tetragonolobus Lectin I (PTL-I), Sophora Japonica Agglutinin (SJA), Peanut Agglutinin (PNA), Euonymus Europaeus Lectin (EEL), Aleuria Aurantia Lectin (AAL), Lotus Tetragonolobus Lectin (LTL), Maclura Pomifera Lectin (MPL), Lycopersicon Esculentum (Tomato) Lectin (LEL), Griffonia (Bandeiraea) Simplicifolia Lectin I (GSL-I), Dolichos Biflorus Agglutinin (DBA), Lens Culinaris Agglutinin (LCA), Ricinus Communis Agglutinin I (RCA120), Solanum Tuberosum (Potato) Lectin (STL), Bandeiraea simplicifolia (BS-I), Canavalia ensiformis (ConA), Psophocarpus Tetragonolobus Lectin II (PTL-II), Datura stramonium (DSA), Soybean Agglutinin (SBA), Vicia Villosa Lectin (VVA), Narcissus Pseudonarcissus Lectin (NPL), Pisum Sativum Agglutinin (PSA), Amaranthus caudatus (ACA), Triticum vulgaris (WGA), Ulex Europaeus Agglutinin I (UEA-I), Phytolacca americana (PWM), Maackia Amurensis Lectin I (MAL-I), Galanthus nivalis (GNA), Bauhinia Purpurea Lectin (BPL), Phaseolus vulgaris Agglutinin (E+L) (PHA-E+L) and Sambucus Nigra Lectin (SNA) were purchased from vector laboratories, Sigma-Aldrich and Calbiochem (Merck), respectively.
